# Author Correction: Remote sensing quantifies widespread abundance of permafrost region disturbances across the Arctic and Subarctic

**DOI:** 10.1038/s41467-019-08375-y

**Published:** 2019-01-23

**Authors:** I. Nitze, G. Grosse, B. M. Jones, V. E. Romanovsky, J. Boike

**Affiliations:** 1 Alfred Wegener Institute, Helmholtz Centre for Polar and Marine Research, 14473 Potsdam, Germany; 2 Institute of Earth and Environmental Science, University of Potsdam, 14476 Potsdam, Germany; 3 Institute of Northern Engineering, University of Alaska Fairbanks, Fairbanks, Alaska 99775 USA; 4 Geophysical Institute, University of Alaska Fairbanks, Fairbanks, Alaska 99775 USA; 5 Department of Cryosophy, Tyumen State University, Tyumen, Russian Federation 625000; 6 Geography Department, Humboldt-Universität zu Berlin, 10099 Berlin, Germany

Correction to: *Nature Communications;*, 10.1038/s41467-018-07663-3; published online 21 December 2018

The original version of this Article contained an error in the author affiliations.

Affiliation 5 incorrectly read ‘Tyumen State Oil and Gas University, Tyumen, Tyument. Oblast, Russian Federation, 625000’.

This has now been corrected in both the PDF and HTML versions of the Article.

