# Supplementary material for: Remote sensing quantifies widespread abundance of permafrost region disturbances across the Arctic and Subarctic
Source: Nat Commun. 2018 Dec 21;9:5423. doi: 10.1038/s41467-018-07663-3 (PMC6303350; doi:10.1038/s41467-018-07663-3)
Supplement: Supplementary file 1 — Supplementary Information [file 41467_2018_7663_MOESM1_ESM.pdf]

Supplementary Information for:

Remote sensing quantifies widespread abundance of permafrost region disturbances across the Arctic and Subarctic

I. Nitze et al.

Supplementary Note 1: Study sites

The western Siberian Transect T1 covers all four permafrost extent zonations from continuous to isolated as well as non-permafrost regions and is predominantly set in sedimentary surface geology consisting of glaciomarine, periglacial and organic deposits with ice contents ranging from medium to high. The Yamal Peninsula, a core region of this transect, was glaciated until around 70-80 ka BP [1] and field and remote sensing studies point out the presence of massive buried ground ice in this region [2]. Another major portion of the transect covers the West Siberian lowlands, a very large wetland with extensive peatland development of global importance for carbon cycling [3]. The T1 transect is characterized by an abundance of thermokarst lakes on the Yamal Peninsula [4], but also in the West Siberian Peatlands [5]. In addition, both regions are currently undergoing heavy economic development from oil and gas activities.

The East-Siberian Transect T2 stretches from the Lena Delta towards central Yakutia and is located nearly completely in the Continuous Permafrost Zone with predominantly high ice-content in low-lying basins. Beside the deltaic and fluvial deposits with variable ice contents in the Lena valley and delta, Yedoma permafrost is widespread in this transect and forms an important and vulnerable reservoir of permafrost carbon [6], where thermokarst lakes are also abundant [7]. Another portion of the transect includes the Verkhoyansk mountain range, which stretches in N-S direction along the eastern banks of the Lena River and has been partially glaciated during the Quaternary [8] (Stauch & Lehmkuhl, 2010). While the surface geology of this mountain range is dominated by ice-poor exposed bedrock, lower slopes and valleys may be blanketed with glaciofluvial, eolian, and periglacial deposits that can be very ice-rich in places. The T2 transect includes Yakutsk, with about 200,000 inhabitants the largest city built on continuous permafrost and the central Yakutia area that is influenced by agricultural practices [9], which also has led to rapid human-caused thermokarst lake formation in the past [10].

Transect T3 encompasses large parts of northern, western and central Alaska and the northwestern tip of Canada. It covers a variety of permafrost types, which have a range of different ice-contents. The region is characterized by mountain ranges, coastal plains and large river deltas, and inland basins. The Arctic coastal plain and foothills are dominated by ice-rich deposits of marine, eolian, and periglacial origins. Thermokarst lakes are widespread in the coastal plains, river deltas, and inland basins [11]. The Brooks Range in northern Alaska, the Akhlun Mountains, and small portions of the mountains on the Seward Peninsula have been partially affected by late Pleistocene glaciation [12]. Again, their foothills and valleys are blanketed by glacial and/or periglacial sedimentary deposits that have high ground ice-contents.

Transect T4 is located along the eastern shore of Hudson Bay in northern Quebec, Canada. It is located on the Canadian Shield with predominantly bedrock geology and low ground ice content. The transect covers the entire range from Continuous to Isolated Permafrost. The entire region was glaciated during the last glacial maximum (~21,000 years ago). While lakes are abundant, many are not thermokarst lakes but lakes situated in glacially carved bedrock depressions. The region is characterized by ongoing rapid isostatic rebound since the Laurentide Ice Sheet had its center in the Hudson Bay region with the last glacial ice disappearing here only during the early Holocene [13].

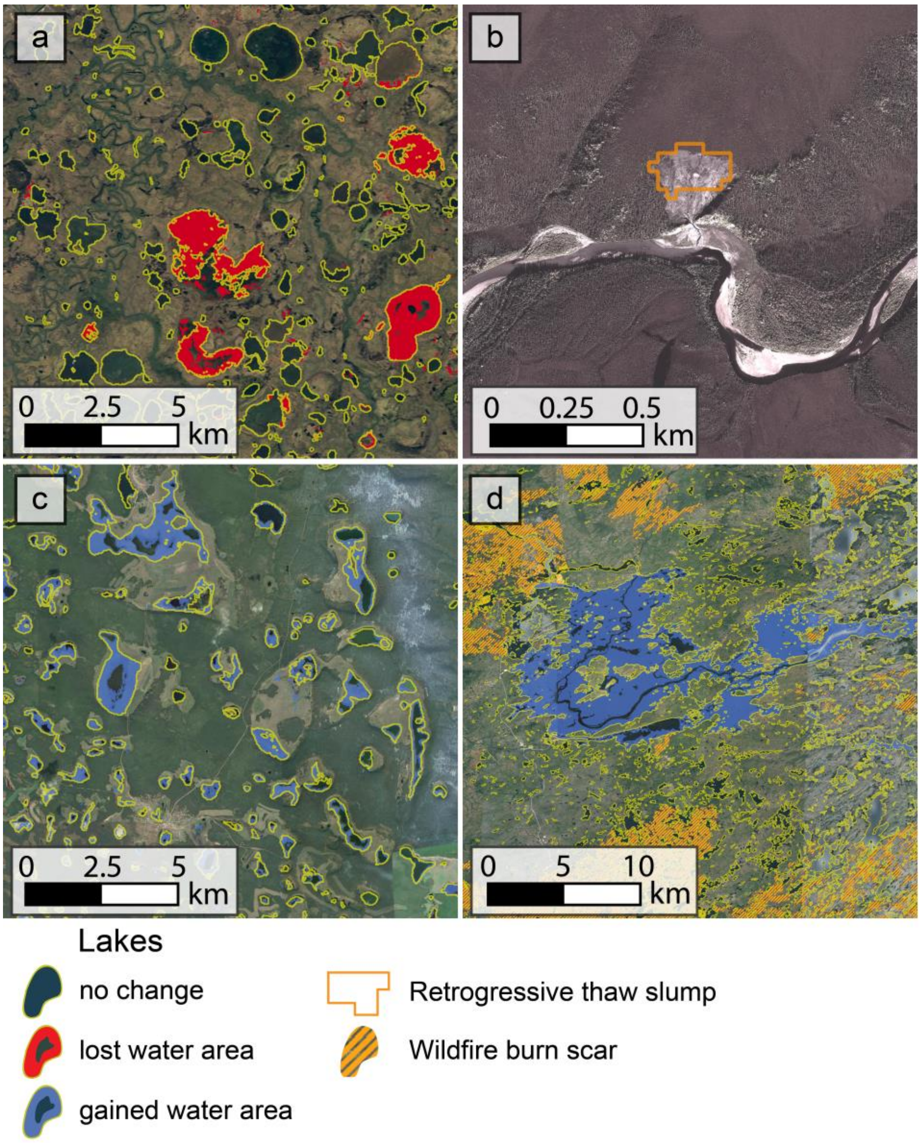

Supplementary Figure 1: Local examples of permafrost related disturbances within each study transect

a) Widespread lake drainage on the southern Yamal Peninsula in western Siberia (T1); b) Selawik slump in Alaska (T3) with detected perimeter of disturbed land surface; c) Widespread lake expansion in Central Yakutia (T2); and d) Filling of Eastmain-1 reservoir and widespread burn scars in Eastern Canada (T4). Approximate Coordinates of locations: a) 69.1°N, 72.05°E; b) 66.5°N, 157.61°W; c) 63°N, 130.55°E; d) 52.1°N; 75.8°W. Background images a-d): Google Satellite layer accessed through QGIS QuickMapServices plugin.

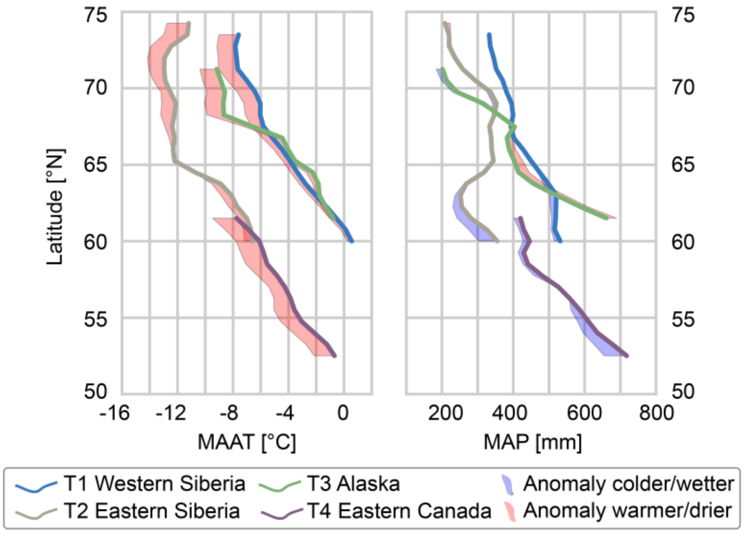

Supplementary Figure 2: Latitudinal climatic parameters of study sites.

Mean annual air temperature (MAAT) and mean annual precipitation (MAP) of study sites during the observation period (1999-2014) and its anomalies to period from 1979 to 1998 calculated from ERA-interim reanalysis data.

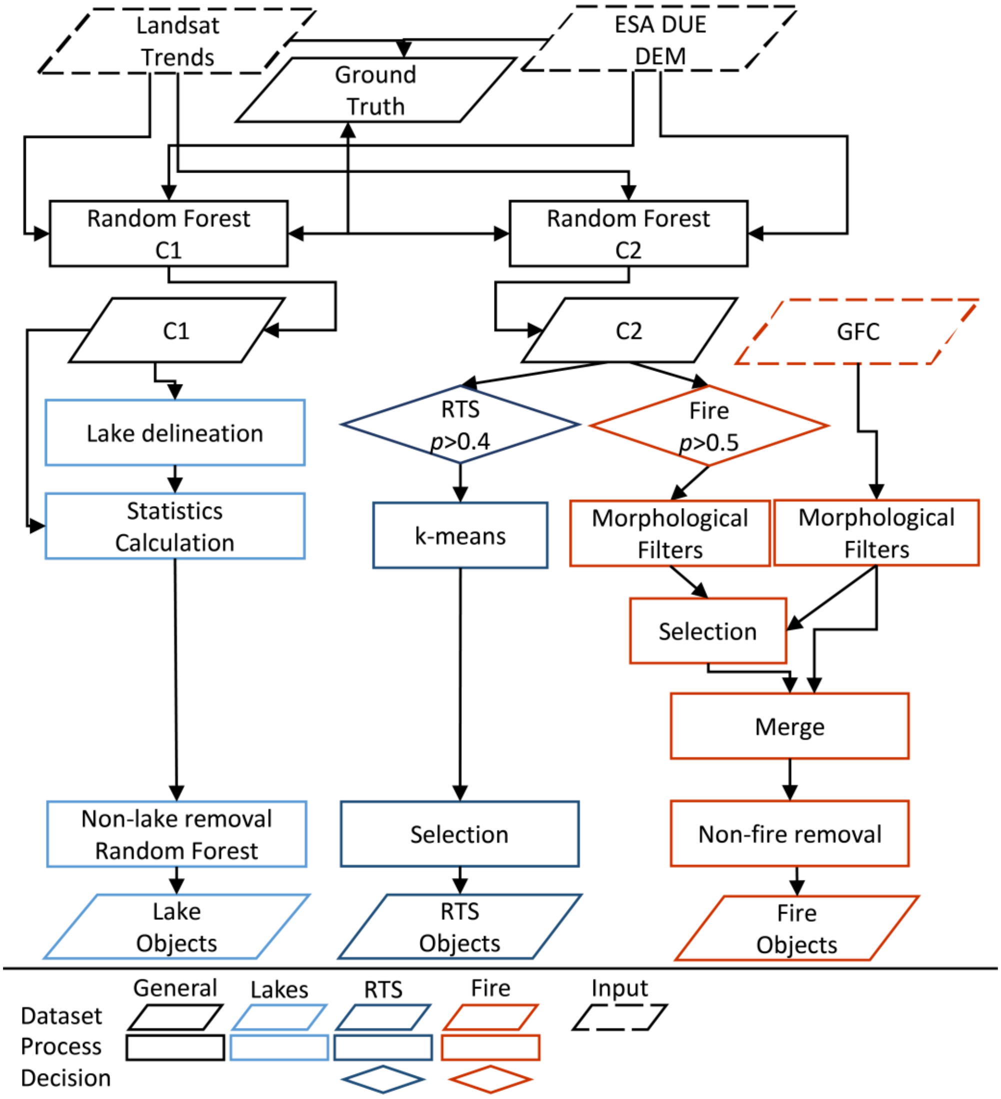

Supplementary Figure 3: Flowchart of PRD classification and object detection workflow.

GFC: Global Forest Change Dataset.

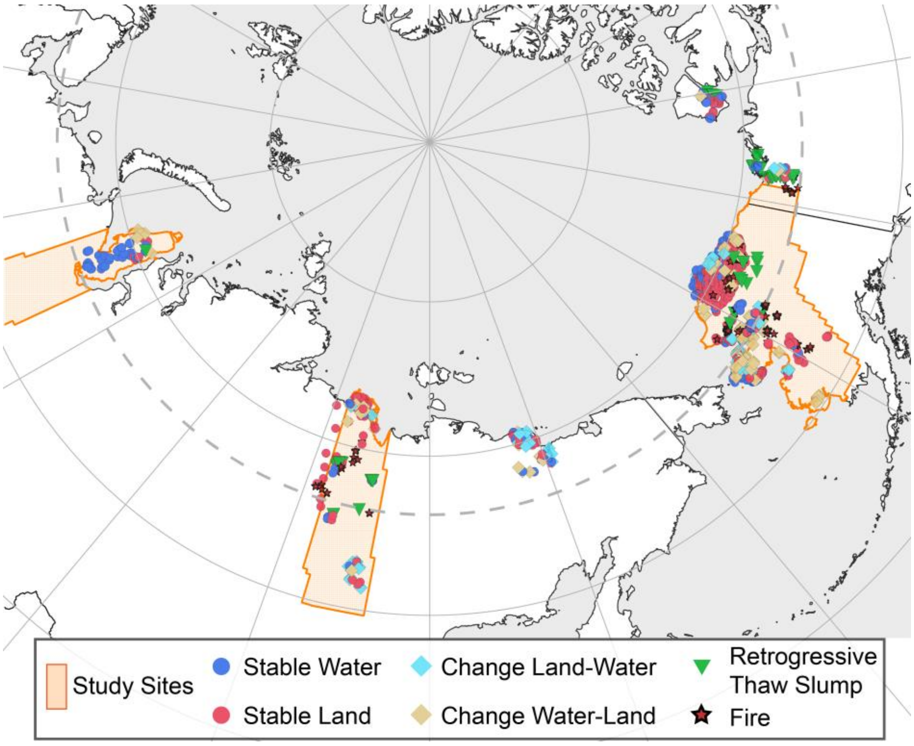

Supplementary Figure 4: Overview of training sample locations for land change process classification.

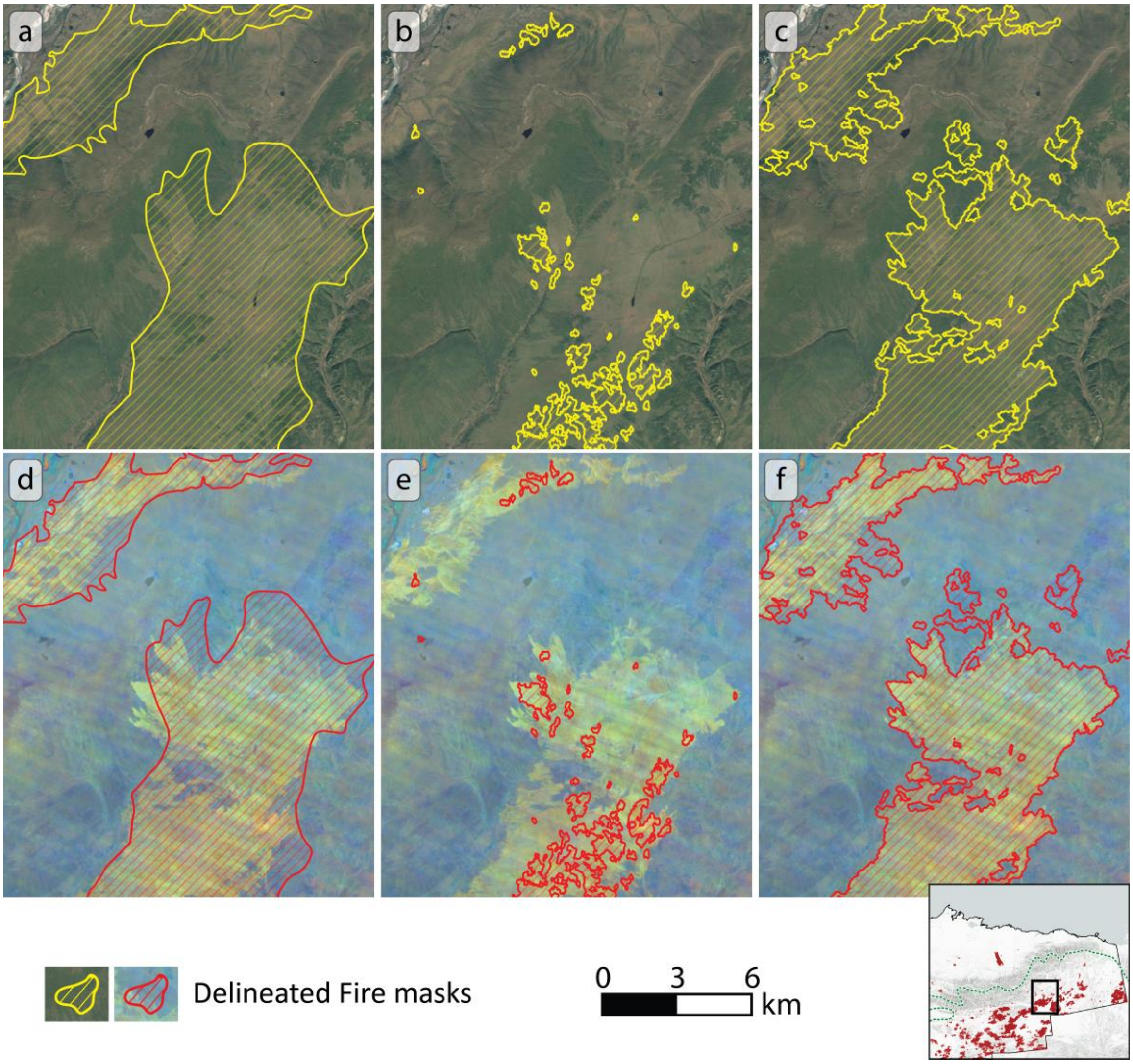

Supplementary Figure 5: Footprint of different fire masks in forest tundra in Alaska.

Fire masks in Forest Tundra in Alaska overlain on True color satellite image background (a-c) and Landsat Trend Analysis Tasseled Cap visualization (d-f). a, d): Alaska Fire Perimeter Dataset; b, e): Fire mask based on Global Forest Change dataset (GFC); c, f): Fire mask based on Landsat Trend Analysis (TBFM). Approximate Coordinates of location: 67.4°N, 146.05°W. Background images a-c): Google Satellite layer accessed through QGIS QuickMapServices plugin. Background images d-f): Landsat Trend Analysis Tasseled Cap Visual Product [14].

Supplementary Table 1: Overview of land cover and change classification with classes, number of reference locations and configuration.

Land cover/change class name	# of reference locations	Class. Config.
Stable Water	299	1
Stable Land	403	1
Change Land to Water	85	1
Change Water to Land	186	1
Wildfire (tundra and boreal)	201	1, 2
Retrogressive Thaw Slump	80	1, 2

Supplementary References

1. Svendsen, J. I. *et al.*, Late Quaternary ice sheet history of northern Eurasia. *Quaternary Science Reviews* **23**, 1229-1271 (2004).

2. Leibman, M. O., Cryogenic landslides on the Yamal Peninsula, Russia: preliminary observations. *Permafrost and Periglacial Processes* **6**, 259-264 (1995).

3. Smith, L. C. *et al.*, Siberian peatlands a net carbon sink and global methane source since the early Holocene. *Science* **303**, 353-356 (2004).

4. Dvornikov, Y. *et al.*, Terrestrial CDOM in Lakes of Yamal Peninsula: Connection to Lake and Lake Catchment Properties. *Remote Sensing* **10**, 167 (2018).

5. Manasypov, R. M., Pokrovsky, O. S., Kirpotin, S. N. & Shirokova, L. S., Thermokarst lake waters across the permafrost zones of western Siberia. *The Cryosphere* **8**, 1177-1193 (2014).

6. Strauss, J. *et al.*, The deep permafrost carbon pool of the Yedoma region in Siberia and Alaska. *Geophysical Research Letters* **40**, 6165-6170 (2013).

7. Morgenstern, A., Grosse, G., Günther, F., Fedorova, I. & Schirrmeister, L., Spatial analyses of thermokarst lakes and basins in Yedoma landscapes of the Lena Delta. *The Cryosphere* **5**, 849-867 (2011).

8. Stauch, G. & Lehmkuhl, F., Quaternary glaciations in the Verkhoyansk Mountains, northeast Siberia. *Quaternary Research* **74**, 145-155 (2010).

9. Crate, S. *et al.*, Permafrost livelihoods: A transdisciplinary review and analysis of thermokarst-based systems of indigenous land use. *Anthropocene* **18**, 89-104 (2017).

10. Fedorov, A. N. *et al.*, Estimating the water balance of a thermokarst lake in the middle of the Lena River basin, eastern Siberia. *Ecohydrology* **7**, 188-196 (2014).

11. Arp, C. D. & Jones, B. M., Tech. rep., 2009.

12. Kaufman, D. S. & Manley, W. F., Pleistocene maximum and Late Wisconsinan glacier extents across Alaska, USA. *Developments in Quaternary Sciences* **2**, 9-27 (2004).

13. Dyke, A., Late Quaternary vegetation history of northern North America based on pollen, macrofossil, and faunal remains. *Geographie physique et Quaternaire* **59**, 211-262 (2005).

14. Nitze, I., in *Trends of land surface change from Landsat time-series 1999-2014* (PANGAEA, 2018).
